# Numerical Abstraction in Young Domestic Chicks (*Gallus gallus*)

**DOI:** 10.1371/journal.pone.0065262

**Published:** 2013-06-11

**Authors:** Rosa Rugani, Giorgio Vallortigara, Lucia Regolin

**Affiliations:** 1 Department of General Psychology, University of Padova, Padova, Italy; 2 Center for Mind/Brain Sciences, University of Trento, Rovereto (Trento), Italy; Université Pierre et Marie Curie, France

## Abstract

In a variety of circumstances animals can represent numerical values *per se*, although it is unclear how salient numbers are relative to non-numerical properties. The question is then: are numbers intrinsically distinguished or are they processed as a last resort only when no other properties differentiate stimuli? The last resort hypothesis is supported by findings pertaining to animal studies characterized by extensive training procedures. Animals may, nevertheless, spontaneously and routinely discriminate numerical attributes in their natural habitat, but data available on spontaneous numerical competence usually emerge from studies not disentangling numerical from quantitative cues. In the study being outlined here, we tested animals' discrimination of a large number of elements utilizing a paradigm that did not require any training procedures. During rearing, newborn chicks were presented with two stimuli, each characterized by a different number of heterogeneous (for colour, size and shape) elements and food was found in proximity of one of the two stimuli. At testing 3 day-old chicks were presented with stimuli depicting novel elements (for colour, size and shape) representing either the numerosity associated or not associated with food. The chicks approached the number associated with food in the 5vs.10 and 10vs.20 comparisons both when quantitative cues were unavailable (stimuli were of random sizes) or being controlled. The findings emerging from the study support the hypothesis that numbers are salient information promptly processed even by very young animals.

## Introduction

A wealth of data has demonstrated that a variety of non-human animals are able to solve some numerical tasks [1], but little is known about how salient numbers are relative to other properties [2]. Some authors have argued that non-human animals can learn to master abstract numerical competence outside of their natural environment only after undergoing extensive laboratory training, which occurs whenever the stimuli employed do not offer quantitative non-numerical information [3], [4], [5], [6], [7]. After being trained to respond to ordinal relationships linked to six Arabic numerical symbols, when tested, squirrel monkeys chose the larger number in all the previously experienced combinations as well as in new ones [8]. Trained to sort in ascending order stimuli representing from 1 to 4 elements, rhesus monkeys [9], hamadryas baboons, squirrel monkeys [10] and brown capuchin monkeys [11] were able to generalize to new numbers –from 5 to 9- and new stimuli. Monkeys trained to respond (in ascending or descending order) to pairs of numerosities (1–9) spontaneously ordered in that same direction new pairs of larger values (i.e., 10,15,20,30) [12]. When trained to place values (6-5-4) in descending order, rhesus macaques were able to apply the descending rule to novel values (1-2-3) [12]. Lemurs (*Lemur catta* and *Eulemur mongoz*), when trained to select between two images the higher-ranking one, could learn the ordinal relationship between seven stimuli, showing a transitive inference reasoning [13]. But abstraction is not just a prerogative of primates: an African grey parrot (*Psittacus erithacus*) was, for example, able to use labels to order numbers from 1 to 8 [14]. Adult Clarks' Nutcrackers could identify the 4^th^ or the 6^th^ positions in a series of 16 identical ones [15]. When all other spatial cues were being controlled, day-old domestic chicks (*Gallus gallus*) could identify an element within a series of identical ones solely on the basis of ordinal information [16], [17]. While these and other studies prove that some animals possess some abstract numerical competence, it cannot be excluded that pure numerical ability emerges only following long laboratory training.

Studies which were, instead, carried out to specifically investigate spontaneous, i.e. in the absence of training, numerical discrimination have been unable to clarify if and when non-verbal subjects (non-human animals and pre-verbal infants) rely on number or on other cues. Many of those studies were performed considering quantitative variables (usually either cumulative surface area or total volume) one at a time [18], [19], [20]. If indeed the quantitative cues are not specifically controlled it becomes impossible to verify if the subjects are relying on quantitative cues or on the number itself [1], [21], [22]. Using a preferential looking method, six-month-old infants discriminated large numerosities that differ by a ratio of 0.5 (8vs.16 or 16vs.32), but not 0.667 (8vs.12 or 16vs.24), when presented with visual arrays in which many quantitative variables were controlled [23], [24]. At the same age infants discriminated 8vs.16 and 4vs.8 sounds but failed in discriminating 8vs.12 and 4vs.6 sounds, providing evidence that the same ratio (0.5) limits numerosity discrimination in auditory-temporal sequences and visuo-spatial arrays [25], [26]. When monkeys (*Macaca mulatta*) observed an experimenter hiding a number or apple pieces, one at a time, in an opaque container and a different number of apple pieces in another opaque container, they approached the larger quantity when the following pairs were presented: 1vs.2, 2vs.3, 3vs.4 and 3vs.5. In order to examine the possibility that the monkeys were focusing on volume rather than on number, in one control condition the experimenters placed 3 pieces of apple in one opaque container and 1 piece of apple, equal in volume to the three pieces grouped together, in another one. The monkeys once again chose the larger number, showing that the numerical cue was considered more important than the quantitative one [27]. Horses (*Equus caballus*), likewise, selected the larger of two quantities when presented with small numerical contrasts (1vs.2 and 2vs.3) even when the total volume of the two sets was equal [28].

Using heterogeneous elements in experimental paradigms seemed to be the best way to effectively control for quantitative variables and consequently to test the abstraction of numerical values. Until now heterogeneous items have been used to test abstract numerical competence in experiments characterized by long training procedures [9], [29], [30]. The study being outlined here describes experiments in which chicks were reared in an environment where food was available only behind a screen picturing a particular number (positive stimulus, i.e. 5) of heterogeneous elements (differing in colour, size and shape) and not behind another screen picturing a different number (neutral stimulus, i.e. 10) of heterogeneous elements. We were interested in investigating the chicks' spontaneous encoding of numerical information during rearing and to evaluating their ability to discriminate between large numbers of heterogeneous items solely on the basis of numerical cues when quantitative variables (area and perimeter) were being controlled.

During testing, the chicks could freely choose to approach the positive or the neutral stimulus. The former pictured the same number of elements as did the positive stimulus but differing for colour, size and shape from the rearing ones and the latter represented the same number of elements as in the neutral stimulus during the rearing period but different again for colour, size and shape. If the animals were spontaneously encoding numerical information, we expected them to move towards the stimulus associated with food both when quantitative cues were missing (due to the use of randomly sized heterogeneous stimuli) and when the cues were not the same as those used during rearing.

## Materials and Methods

### Ethics Statement

The experiments complied with all applicable national and European laws concerning the use of animals in research and were approved by the Italian Ministry of Health (permit number: 5/2012 emitted on 10/1/2012).

All procedures employed in the experiments included in this study were examined and approved by the Ethical Committee of the University of Padua (Comitato Etico di Ateneo per la Sperimentazione Animale – C.E.A.S.A.) as well as by the Italian National Institute of Health (N.I.H).

### Experiment 1

The goal of the first experiment was to investigate the chicks' ability to discriminate between large numbers of heterogeneous items (5 *vs.*10) solely on the basis of numerical cues (when quantitative variables were unavailable), or when quantitative variables (area and perimeter) are being controlled. Since we were interested in spontaneous encoding of numerical information, the chicks were exposed for about two days to the contingent presentation of food with a certain number (i.e. 5) of items and not with another (i.e. 10). A similar procedure had been used to demonstrate chicks' spontaneous discrimination of possible and impossible objects [31] and their sensitivity to the Ebbinghaus illusion [32] as well as other types of numerical discrimination [33].

#### Subjects

“Hybro” domestic chicks (*Gallus gallus*), a local variety of the White Leghorn breed, were used. These were obtained weekly, every Monday morning when they were a few hours old, from a local commercial hatchery (Agricola Berica, Montegalda, Vicenza, Italy). On arrival, the chicks were housed individually in standard metal cages (28×32×40 cm). Chicks were housed individually as this procedure allowed to employ half of the animals and it allowed to obtain more informative data. In fact data obtained from individual chicks that have been reared in pairs are not independent. Moreover, individual testing would be distressful to pair-reared chicks.

The rearing room was constantly monitored for temperature (28–31°C) and humidity (68%) and was illuminated continuously by fluorescent lamps (36 W) located 45 cm above each cage. Water, placed in transparent glass jars (5 cm in diameter, 5 cm high) in the centre of the cages, was available *ad libitum*.

During the three days, rearing period (from Monday to Wednesday morning), the chicks found food behind two of four vertical plastic screens (10×14 cm) located approximately 10 cm in front of each of the cage's four corners. The two screens hiding food were decorated with identical pictures representing a certain number of elements (*Positive Stimulus*, *S^p^*) while the other two screens not associated with food were decorated with identical pictures of a different numerousness (*Neutral Stimulus*, *S^n^*). All of the screens were covered with static 2D images picturing a certain number of elements whose images were randomly selected from sets of patterns of different shapes (10 different), colours (10 different) and sizes (10 different, ranging between 0.5 cm and 2 cm) and printed on identical white rectangular plastic boards (screens) (11.5×9 cm).

**Figure 1:**
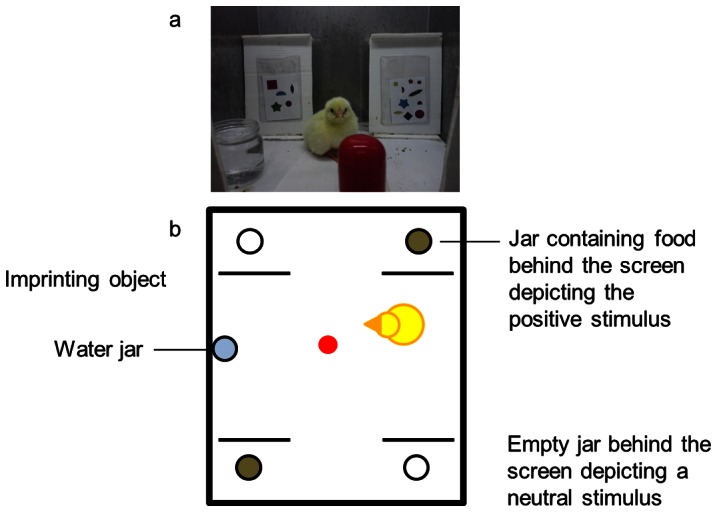
Images of a rearing cage. a) Two screens, each depicting a different number of heterogeneous elements, are visible in the picture. b) A schematic illustration of the rearing cage showing where the screens were located.

During the rearing period four screens were always present in each cage: two representing *S^p^* and two representing *S^n^* (Fig. 1). To prevent the chicks from learning to identify the stimuli on the basis of the spatial disposition of the elements depicted on the screens, six different pairs of stimuli were used. In each, the elements' disposition on the screens was randomly determined in such a way that the distance between elements varied from 0.3 to 3.8 cm. Three times a day the stimuli were replaced, in such a way that each chick was exposed, for about 8 hours, to each pair of stimuli. Every time the stimuli were replaced, the screens were also rotated from corner to corner in order to avoid positional learning. An artificial imprinting object (a red capsule measuring 2×3 cm) was suspended (at chick's height) in each rearing cage to prevent social isolation. Artificial imprinting objects are effective social substitutes of real social mates. After about one-two hours of exposure the chick responds to the artificial object with a range of behavioural responses which are clearly identifiable as social-affiliative [34], [35], [36], [37], [38].

On the morning of the third day (testing day) each chick underwent a single test to verify how the rearing period had affected their numerical discrimination. Numerosities used during testing were the same as those utilized during the rearing period, but all the elements appearing on the screens were new and presented in different spatial dispositions. Three *Testing Conditions*, depending on the dimensions of the stimuli used, were employed. In the Random Size Group (RSG, N = 20, with group-5E: N = 10 and group-10E: N = 10), the dimensions of the elements were randomly presented; in the Perimeter Control Group (PCG, N = 30, with group-5E: N = 15, and group-10E: N = 15) the overall perimeter of the two stimuli was equated; and in the Area Control Group (ACG, N = 40 with group-5E: N = 20 and group-10E: N = 20) the overall areas of the two stimuli were equated.

#### The Apparatus and Procedure

**Figure 2:**
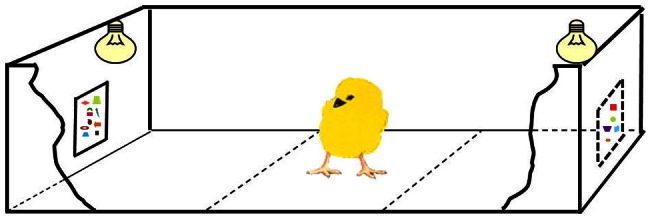
The experimental apparatus. An illustration of the apparatus used in all of the experiments.

Testing took place in an experimental room, adjacent to the rearing room, in which temperature and humidity were controlled (25°C and 70%, respectively) and which was kept dark except for light shining from two lamps (40W) placed at a height of 25 cm at either end of the apparatus. The apparatus (Fig. 2) consisted of a runway (45 cm long, 20 cm wide and 30 cm high). One of the two stimuli was placed at the far end of each side of the apparatus, at a height of 2 cm, so that it was entirely visible to a chick placed in the central area of the apparatus. The positions of the two testing stimuli and the bird's starting position (i.e. with the *S^p^* either to the left or to the right) were balanced across the experiments. The apparatus was made up of three compartments (each 15 cm long): a central, starting area considered a no-choice zone and two side compartments (choice zones).

On testing day, each chick was individually placed at the starting area with the two (positive and neutral) stimuli positioned at the end of the two arms of the apparatus. Choosing one of the two compartments (indicative of its preference for that stimulus) meant that at least ¾ of the chick's body entered the area as the subject was looking at the screen. No choice was instead scored whenever chicks entered a choice zone but looked at the opposite screen [15], [33]. At starting time each chick was placed in the starting position and its behaviour was videorecorded throughout the duration of the experiment, i.e. six minutes. Placed above the apparatus and connected to a monitor, a video camera enabled the experimenter to track the chicks' behaviour during the test without being seen using a computer operated device. This was activated every time a chick entered a choice zone, and registered the amount of time each chick spent near to either stimulus.

An index of choice was calculated for every chick according to the formula used to analyse choice behaviour [39]:

The times spent approaching the *S^p^*/(Time spent near *S^n^* + Time spent near *S^p^*) were calculated. Values at about 0.5 indicated no preference for either stimulus; values >0.5 indicated a preference for *S^p^* and values <0.5 indicated a preference for *S^n^*. Significant differences with respect to chance level (0.5) were calculated by one-sample two tailed t-tests.

#### Results and Discussion

**Figure 3:**
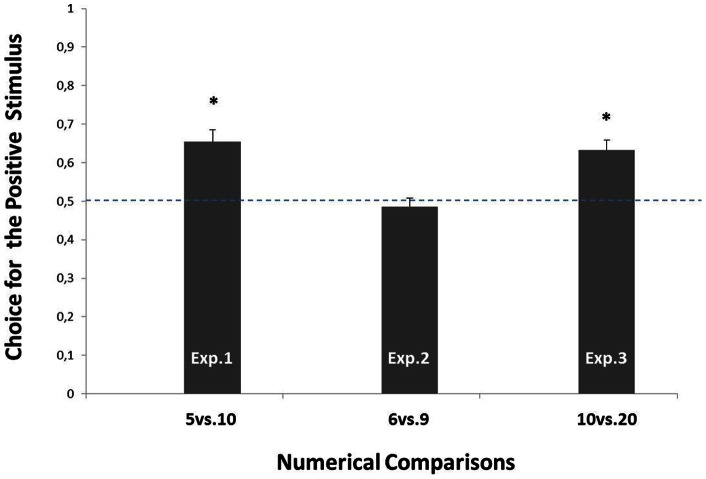
Results of Experiment 1(5vs.10), Experiment 2 (6vs.9) and Experiment 3 (10vs.20). Choice (means with SEM) displayed at testing by the chicks, expressed as a preference for the stimulus associated with food.

An ANOVA was used to analyze *S^p^* (5E or 10E) and the *Testing Condition* (RSG, PCG or ACG) as well as the independent variables. The dependent variable was the *Index of Choice* for *S^p^*. The ANOVA did not uncover a significant main effect for *S^p^* (F(1,84) = 0.192, p = 0.763; group-5E: N = 45; Mean = 0.657, SEM  = 0.031; group-10E: N = 45; Mean  = 0.643, SEM  = 0.023) or for *Testing Condition* (F(2,84) = 0.092; p = 0.763: RSG: N = 20; Mean  = 0.642, SEM  = 0.036; PCG: N = 30; Mean  = 0.673, SEM  = 0.029; ACG: N = 40; Mean  = 0.646, SEM  = 0.028). As the interaction (*Testing Condition* × *S^p^*) was not significant (F(2,84) = 0.270, p = 0.764), the data were merged and the resulting mean (N = 90; Mean  = 0.654, SEM  = 0.031) was found to be significantly above chance level (one-sample t-test, t(89) = 4.968; p<0.001), see Fig. 3.

These results demonstrate that 3-day-old chicks spontaneously discriminate between the numbers of heterogeneous elements even when quantitative variables (area and perimeter) are being controlled. This behaviour seems to be explained by Analogue Magnitude System (AMS) processing, a non-verbal numerical system according to which encoding of numerosities is only approximate [40]. Depending on the ratio between numbers to be discriminated and in accordance with Weber's law, as the ratio between the numerosities to be discriminated becomes larger, the response times decrease and accuracy increases [40].

### Experiment 2

Experiment 1 demonstrated that numerical discrimination of large (5vs.10) sets of heterogeneous elements is possible solely on a numerical basis. In experiment 2 we used a comparison (i.e. 6vs.9) with a 0.67 ratio which is more difficult to discriminate than the previous one (0.50).

#### Subjects, Apparatus and Procedure

A new group of 40 chicks was used. All the chicks were tested using the same numerical comparison: 6vs.9. The rearing conditions were the same as those described above. For one subgroup of chicks (group-6E: N = 20) *S^p^* pictured 6 elements (6E) and for the second (group-9E: N = 20) *S^p^* pictured 9 elements (9E). Six pairs of rearing stimuli differing from one another with regard to the spatial disposition of the elements pictured were used. The testing stimuli were composed of heterogeneous elements positioned in a different way with respect to the rearing situation.

#### Results and Discussion

A t-test comparing the *Index of Choice* registered by the two groups did not reveal any significant difference (t(38) = 1.022; p = 0.313; group-6E: N = 20; Mean  = 0.463, SEM  = 0.034; group-9E: N = 20; Mean  = 0.508, SEM  = 0.028). The data from the two groups were therefore merged, and the resulting mean (N = 40; Mean  = 0.486, SEM  = 0.022) did not result different from chance level (one-sample t-test, t(39) = 0.636; p = 0.528), see Fig. 3.

The first two experiments showed that the chicks were unable to discriminate between two sets of elements characterized by a 0.67 (6vs.9) ratio while they were able to distinguish between two sets characterized by a 0.50 (5vs.10) ratio.

### Experiment 3

The aim of this experiment was to evaluate if there is an absolute upper limit in numerical discrimination. As the 0.50 ratio was discriminated when the 5vs.10 comparison was employed, we arbitrarily decided to duplicate each set: the comparison tested here was therefore 10vs.20.

#### Subjects, Apparatus and Procedure

A new group of 66 chicks was used. The rearing conditions were the same as in the previous experiments. All the chicks were tested using the same numerical comparison: 10vs.20. Rearing stimuli consisted of six pairs each of 10 or 20 heterogeneous elements. The testing stimuli consisted once again of heterogeneous elements, but with different spatial dispositions, colours, shapes and sizes with respect to the rearing stimuli and which varied in the three *Testing Conditions*. In the Random Size Group (RSG, N = 29, with group-10E: N = 14 and group-20E: N = 15) the dimensions of the elements were randomly selected in both the 10E and the 20E. In the Perimeter Control Group (PCG, N = 18, with group-10E: N = 9, and group-20E: N = 9) the overall perimeter of the two stimuli was equated. While in the Area Control Group (ACG, N = 19 with group-10E: N = 10 and group-20E: N = 9) the overall areas of the two stimuli were equated.

#### Results and Discussion

An ANOVA was used to analyse the *S^p^* (10E or 20E) and *Testing Condition* (RSG, PCG or ACG) as well as the independent variables. The dependent variable was the *Index of Choice* for *S^p^*. The ANOVA did not uncover a significant main effect for *S^p^* (F(1,60) = 0.017, p = 0.900; group-10G: N = 33; Mean  = 0.639, SEM  = 0.025; group-20G: N = 33; Mean  = 0.626, SEM  = 0.021) or for *Testing Condition* (F(2,60) = 0.049; p = 0.952: RSG: N = 29; Mean  = 0.627, SEM  = 0.029; PCG: N = 18; Mean  = 0.641, SEM  = 0.025; ACG: N = 18; Mean  = 0.633, SEM = 0.027). As the interaction (*Testing Condition* × *S^p^*) was not significant (F(2,60) = 0.610, p = 0.547), the data were merged and the resulting mean (N = 66; Mean  = 0.633, SEM  = 0.026) was found to be significantly above chance level (one-sample t-test, t(65) = 5.115; p<0.001) (Fig. 3).

## Conclusions

The aim of this study was to test 3-day old chicks' capacity to discriminate between stimuli representing different numerosities and, in particular, to assess their ability to process those stimuli entirely on the basis of numerical cues. The results demonstrate that chicks discriminate between numbers, even when quantitative variables are unavailable, if the numbers compared have a 0.50 ratio (either 5vs.10 or 10vs.20), but not when the ratio between numbers is 0.67 (i.e., 6vs.9). Performance, therefore, seems to be affected not by the overall absolute number of items (15 elements are discernible in the comparisons 5vs.10 but not in the 6vs.9), but by the ratio between the numbers to be discriminated. This suggests that processing in the cases described is carried out using the Analogue Magnitude System (AMS).

Interestingly, these results differ from those outlined in precedent study [41] focusing on the same species but using a different experimental setting; in that case the chicks were able to discriminate both between 5vs.10 and 6vs.9, but only when quantitative as well as numerical cues were available. Why were the chicks investigated during the current study able to discriminate numerosities on the basis of numerical cues alone? In order to answer that question we need to go back and re-examine the 2011 study. During the experiments carried out at that time, the chicks were reared with a set of artificial social objects upon which they became imprinted. Social objects were also employed during the experiments carried out during our latest study, but the ones used (two-dimensional red squares) were all identical to one another during the rearing period while at testing each of the two sets was composed of homogeneous elements (although elements could differ in size to control for quantitative variables). At testing during the 2011 study, the chicks watched the imprinted objects disappearing one at a time (i.e. sequentially) behind screens located in different positions in the test area. After all the elements had appeared and disappeared the chicks were expected to look for the larger set associated to one of the two screens. If we want to compare the two studies, we need to examine how they differed in terms of: i. the characteristics of the stimuli used (homogeneous vs. heterogeneous sets, respectively, in the precedent and in the current study), and ii. the modality in which the chicks experienced the stimuli during the test. i. Stimuli characteristics (homogeneous vs. heterogeneous). Some investigators have suggested that using sets of objects made up of elements identical to one other (i.e., a homogenous set) favours computation of continuous variables [42], [43], [44], [45], while heterogeneity of objects within the same set favours computation of exact numbers [46], [47], [48]. In accordance with these hypotheses, our precedent results indicated that when the chicks were required to discriminate between homogeneous sets of items in a 2vs.3 comparison, they processed quantity information, but when heterogeneous stimuli were presented, the chicks coded the numerousness [49]. ii. Stimuli presentation at testing. When stimuli disappear one after another, as in our sequential presentation paradigm [29], higher demands are posed on the working memory since they are no longer perceptually available at the moment of choice; a lower performance is thus expected. The modality of stimulus presentation could, according to a recently proposed hypothesis [50] and verified by different brain activation patterns, favours diversified kinds of elaboration. According to that hypothesis, the simultaneous presentation of whole sets of elements directs attention to the entire collection, thus activating AMS processing. On the contrary, presenting the elements one after another, focuses attention on each object, thus activating the Object File System (OFS) processing which concentrates on single objects at the expense of the overall set, which in any case cannot contain more than 3 elements [51]. These considerations appear relevant to the results emerging from our two studies; in fact in the present study the whole sets of elements were visibly accessible to the chicks during testing, while in the 2011 paradigm the stimuli used at testing were presented sequentially. It is important to underline that the difference in the two studies with regard to stimuli presentation was limited to the testing phase. During the rearing stage the chicks in both studies were exposed to the entire sets of stimuli, probably triggering AMS processing. There may have been interference in the cognitive systems activated during the 2011 study when sequential presentation (linked to OFS) was used at testing. It would be interesting in future studies to directly test how stimuli presentation during rearing with respect to testing stages can affect OFS or AMS processing.

Some considerations can also been drawn regarding the paradigm used in the current study that made it possible to highlight the chicks' spontaneous learning and therefore to better assess their spontaneous behaviour. After rearing, the chicks, in fact, associated some numerical patterns with food. In contrast with previous studies [39], learning did not require any explicit training (i.e. through shaping) during which an operant behavioural response (i.e. pecking at the positive stimulus) was reinforced as in conventional discrimination learning tasks. Our paradigm offers an advantage with respect to tasks requiring animals to choose/directly respond to two or more food options. In fact, animals' choices in those cases are expected to maximize possibility of survival according to the optimal foraging theory [52]. Researchers carrying out numerical discrimination tasks [53], [54], [55], [56], [57], [58] consider preference/choice directed towards the larger quantities of food a strategy directed at exploiting food resources. Although this is often the case, the best choice could also depend on a variety of factors – usually neglected – but not necessarily related to overall amounts such as optimal size of food for catching or ingesting, optimal density, etc. Differences in spontaneous discrimination in diverse species could then be related to their foraging strategy rather than to numerical cognitive skills. Direct comparison between food quantities generally cannot guarantee simultaneous control over quantitative variables, such as volume, surface, etc. of food items. In those cases data on spontaneous foraging demonstrate spontaneous proto-numerical discrimination, but do/can not test purely numerical abilities. The paradigm used in the current study offers an advantage also with respect to conventional operant conditioning, which is known to be linked to a behavioural response between the stimulus and the reinforcement. It is well known that when an auto-shaping paradigm is used, pigeons auto-reinforced with water manifest a drinking behaviour to “previous neutral stimulus” (a key), while those auto-reinforced with food show an eating behaviour [59]. The concept of positive behavioural reinforcement could be extended to paradigms using shaping to test numerical competences and help explain some results. For example, pigeons trained to discriminate between two numerousness of non-edible elements to obtain a food reinforcement performed better when reinforced to respond to the larger rather than to the smaller numerousness [60]. By contrast, the performance of the chicks' studied here did not differ when food was associated with a larger or a smaller number of objects, and this could be explained by the fact that no conditioned pecking response was required for the stimulus to be associated with food. Under these conditions the characteristics of the stimulus (numerousness of objects) are not affected by the features of the attractor (food), thus making it possible to manipulate the latter as required and to better investigate numerical cognitive abilities. The same paradigm could easily be employed using a different kind of attractor, i.e., a social (i.e. an imprinting) object in order to further reduce any possible association between food and stimulus.

To conclude, this work demonstrated how an abstract recognition of large [16] numbers could be precociously available in one animal species, disproving the ‘last resort’ theory, according to which animals rely on numerical information solely when quantitative cues (considered more salient) are not available [61]. The data outlined here support the hypothesis that animals naturally extrapolate and use numerical cues, suggesting that numerical information constitutes a crucial cue for animal survival.

## References

[1] Vallortigara G, Chiandetti C, Sovrano VA, Rugani R, Regolin L (2010) Animal Cognition. Wiley Interdisciplinary Reviews: Cogn Sci 1: 882–893. 10.1002/wcs.75 26271784

[2] Cantlon JF, Brannon EM (2007) How Much Does Number Matter to a Monkey (*Macaca mulatta*)? J Exp Psychol: Anim Behav Proc 33(1): 32–41. 10.1037/0097-7403.33.1.32 17227193

[3] Breukelaar JWC, Dalrymple-Alford JC (1998) Timing ability and numerical competence in rats. J Exp Psychol: Anim Behav Proc 24: 84–97. 10.1037//0097-7403.24.1.84 9438968

[4] Davis H (1993) Numerical competence in animals: Life beyond Clever Hans. In S. T. Boysen & E. J. Capaldi (Eds.), The development of numerical competence: Animal and human models (109–125). Hillsdale, NJ: Erlbaum.

[5] Davis H, Memmott J (1982) Counting behavior in animals: A critical evaluation. Psychol Bul 92: 547–571.

[6] Davis H, Pérusse R (1988) Numerical competence in animals: definitional issues, current evidence, and new research agenda. Behav Brain Sci 11: 561–615.

[7] Seron X, Pesenti M (2001) The Number Sense Theory Needs More Empirical Evidence Mind and Language. 16(1): 76–88.

[8] Olthof A, Iden CM, Roberts WA (1997) Judgments of ordinality and summation of number symbols by squirrel monkeys (*Saimiri sciureus*). J Exp Psychol: Anim Behav Proc 23: 325–333. 10.1037//0097-7403.23.3.325 9206027

[9] Brannon EM, Terrace HS (1998) Ordering of the numerosities 1 to 9 by monkeys. Science 282(5389): 746–749. 9784133 10.1126/science.282.5389.746

[10] Smith BR, Piel AK, Candland DK (2003) Numerity of a socially housed hamadryas baboon (*Papio hamadryas*) and a socially housed squirrel monkey (*Saimiri sciureus*). J Comp Psychol 117(2): 217–225. 12856792 10.1037/0735-7036.117.2.217

[11] Judge PG, Evans TA, Vyas TK (2005) Ordinal representation of numeric quantities by brown capuchin monkeys (*Cebus apella*). J Exp Psychol: Anim Behav Proc 31(1): 79–94. 10.1037/0097-7403.31.1.79 15656729

[12] Cantlon J, Brannon EM (2006) The effect of heterogeneity on numerical ordering in rhesus monkeys. Infancy 9(2): 173–189.

[13] McLean E, Merritt DJ, Brannon ME (2008) Social complexity predicts transitive reasoning in prosiamian primates. Anim Behav 76: 479–486. 19649139 10.1016/j.anbehav.2008.01.025 PMC2598410

[14] Pepperberg MI (2012) Further evidence for addition and numerical competence by a Grey parrot (*Psittacus erithacus*). Anim Cogn doi: 10.1007/s10071-012-0470-5. 10.1007/s10071-012-0470-5 22402776

[15] Rugani R, Kelly MD, Szelest I, Regolin L, Vallortigara G (2010) It is only humans that count from left to right? Biol Lett 6: 290–292. 20071393 10.1098/rsbl.2009.0960 PMC2880063

[16] Rugani R, Regolin L, Vallortigara G (2007) Rudimental competence in 5-day-old domestic chicks: Identification of ordinal position. J Exp Psychol: Anim Behav Proc 33(1): 21–31. 10.1037/0097-7403.33.1.21 17227192

[17] Rugani R, Vallortigara G, Vallini B, Regolin L (2011) Asymmetrical number-space mapping in the avian brain. Neurobiol Learn Mem 95: 231–238. 21111840 10.1016/j.nlm.2010.11.012

[18] Boysen ST, Berntson GG (1989) Numerical competence in a chimpanzee (*Pan troglodytes*). J Comp Psychol 103: 23–31. 2924529 10.1037/0735-7036.103.1.23

[19] Beran MJ (2001) Summation and numerousness judgments of sequentially presented sets of items by chimpanzees (*Pan troglodytes*). J Comp Psychol 115: 181–191. 11459165 10.1037/0735-7036.115.2.181

[20] Beran MJ, Washburn DA, Smith JD, Redford JS (2006) Rhesus macaques (*Macaca mulatta*) monitor uncertainty during numerosity judgments. J Exp Psychol: Anim Behav Proc 32: 111–119. 10.1037/0097-7403.32.2.111 16634654

[21] Vallortigara G, Chiandetti C, Sovrano VA, Rugani R, Regolin L (2010) Animal Cognition. Wiley Interdisciplinary Reviews: Cognitive Science 1: 882–893. 26271784 10.1002/wcs.75

[22] Backer JM, Morath JM, Rodzon KS, Jordan KE (2012) A shared system of representation governing quantity discrimination in canids. Frontiers in Psychol doi: 10.3389/fpsyg.2012.00387. 10.3389/fpsyg.2012.00387 PMC3465982 23060847

[23] Xu Spelke (2000) Large number discrimination in 6-month-old infants. Cognition: 74, B1–B11. 10.1016/s0010-0277(99)00066-9 10594312

[24] Xu F, Spelke ES, Gottard S (2005) Number sense in human infants. Dev Sci 8(1): 88–101. 15647069 10.1111/j.1467-7687.2005.00395.x

[25] Lipton JS, Splelke ES (2004) Discrimination of large and small numerosities by human infants. Infancy 5(3): 271–290.

[26] Lipton JS, Spelke ES (2005) Origins of number sense: Large-number discrimination in human infants. Psychol Sci 14(5): 396–401. 10.1111/1467-9280.01453 12930467

[27] Hauser MD, Carey S, Hauser L (2000) Spontaneous number representation in semi-free-ranging rhesus monkeys. Proc R Soc Lond B 267: 829–833. 10.1098/rspb.2000.1078 PMC1690599 10819154

[28] Uller C, Lewis J (2009) Horses (*Equus caballus*) select the greater of two quantities in small numerical contrasts. Anim Cogn 12: 733–738. 19387706 10.1007/s10071-009-0225-0

[29] Merritt DJ, Rugani R, Brannon EM (2009) Empty sets as part of the numerical continuum: conceptual precursors to the zero concept in rhesus monkeys. J Exp Psychol Gen 138(2): 258–269. 19397383 10.1037/a0015231 PMC2918401

[30] Scarf D, Hayne H, Colombo M (2011) Pigeons on par with primates in numerical competence. Science 334: 1664. 22194568 10.1126/science.1213357

[31] Regolin L, Rugani R, Stancher G, Vallortigara G (2011) Spontaneous discrimination of possible and impossible objects by newly hatched chicks. Bio Let 7: 654–657. 21429912 10.1098/rsbl.2011.0051 PMC3169041

[32] Rosa Salva O, Rugani R, Cavazzana A, Regolin L, Vallortigara G (2013) Perception of Ebbinghaus illusion in domestic chick (*Gallus gallus*). Anim Cogn doi: 10.1007/s10071-013-0622-2. 10.1007/s10071-013-0622-2 23572064

[33] Rugani R, Regolin L, Vallortigara G (2013). One, two, three, four, or is there something more? Numerical discrimination in day-old domestic chicks. Anim Cogn doi: 10.1007/s10071-012-0593-8. 10.1007/s10071-012-0593-8 23334508

[34] Bolhuis JJ (1991) Mechanism of avian imprinting. Bio Rev 66: 303–345. 1801945 10.1111/j.1469-185x.1991.tb01145.x

[35] Bateson P (2000) What must be known in order to understand imprinting? ln Heyes, C. & Huber, 1., eds. The Evolution of Cognition. 85–102. Cambridge, Mass: The MIT Press.

[36] Regolin L, Rugani R, Pagni P, Vallortigara G (2005) Delayed search for a social and a non-social goal object by the young domestic chick (*Gallus gallus*). Anim Behav 70: 855–864.

[37] Regolin L, Garzotto B, Rugani R, Vallortigara G (2005) Working memory in the chick: parallel and lateralized mechanisms for encoding of object- and position- specific information. Behav Brain Res 1(57): 1–9. 10.1016/j.bbr.2004.06.012 15617765

[38] Fontanari L, Rugani R, Regolin L, Vallortigara G (2011) Object individuation in three-day old chicks: Use of property and spatiotemporal information. Dev Sci 14(5): 1235–1244. 21884338 10.1111/j.1467-7687.2011.01074.x

[39] Andrew RJ (1991) The nature of behavioural lateralization in the chick. In: Andrew RJ, editor. Neural and behavioural plasticity: the use of the chick as a model. Oxford: Oxford University Press: 536–54.

[40] Gallistel CR, Gelman R (1992) Preverbal and verbal counting and computation. Cognition 44: 43–74. 1511586 10.1016/0010-0277(92)90050-r

[41] Rugani R, Regolin L, Vallortigara G (2011) Summation of large numerousness by newborn chicks. Frontiers Comp Psychol; Online First doi: 10.3389/fpsyg.2011.00179. 10.3389/fpsyg.2011.00179 PMC3171108 21941514

[42] Clearfield MW, Mix KS (1999) Number versus contour length in infant's discrimination of small visual sets. Psychol Sci 10: 408–411.

[43] Clearfield MW, Mix KS (2001) Amount Versus Number: Infants' Use of Area and Contour Length to Discriminate Small Sets. J Cogn Dev 2(3): 243–260.

[44] Feigenson L, Carey S, Spelke E (2002) Infant's discrimination of number versus continuous extent. Cog Psychol: 44, 33–66. 10.1006/cogp.2001.0760 11814309

[45] Feigenson L, Carey S, Hauser M (2002) The Representations underlying infants' choice of more: object files versus analog magnitudes. Psychol Sci13(2): 150–156. 10.1111/1467-9280.00427 11933999

[46] Feigenson L, Carey S, Spelke E (2002) Infant's discrimination of number versus continuous extent. Cogn Psychol 44: 33–66. 11814309 10.1006/cogp.2001.0760

[47] Feigenson L, Carey S (2003) On the limits of infants' quantification of small object arrays. Cognition 97(3): 295–313. 10.1016/j.cognition.2004.09.010 16260263

[48] Feigenson L, Carey S (2005) On the limits of infants' quantification of small object arrays. Cognition 97: 295–313. 16260263 10.1016/j.cognition.2004.09.010

[49] Rugani R, Regolin L, Vallortigara G (2010) Imprinted numbers: newborn chicks' sensitivity to number vs. continuous extent of objects they have been reared with. Dev Sci 13: 790–797. 20712745 10.1111/j.1467-7687.2009.00936.x

[50] Hyde DC, Spelke ES (2011) Neural signatures of number processing in human infants: Evidence for two core systems underlying numerical cognition. Dev Sci 14(2): 360–371. 21399717 10.1111/j.1467-7687.2010.00987.x PMC3050652

[51] Rugani R, Regolin L, Vallortigara G (2008) Discrimination of small numerosities in young chicks. J Exp Psychol: Anim Behav Proc 34(3): 388–399. 10.1037/0097-7403.34.3.388 18665721

[52] Krebs JR (1974) Colonial nesting and social feeding as strategies for exploiting food resources in the great blue heron (Ardea herodias). Behaviour 51: 99–130.

[53] Uller C, Jaeger R, Guidry G (2003) Salamanders (Plethodon cinereus) go for more: rudiments of number in an amphibian. Anim Cogn 6: 105–112. 12709845 10.1007/s10071-003-0167-x

[54] Call J (2000) Estimating and operating on discrete quantities in oranges (*Pongo pygmaeus*). J Comp Psychol 114: 136–147. 10890585 10.1037/0735-7036.114.2.136

[55] Ward C, Smuts BB (2007) Quantity-based judgments in the domestic dog (*Canis lupus familiaris*). Anim Cog 10(1): 71–80. 10.1007/s10071-006-0042-7 16941158

[56] Irie-Sugimoto N, Kobayashi T, Sato T, Hasegawa T (2009) Relative quantity judgment by Asian elephants (*Elephas maximus*). Anim Cog 12: 193–199. 10.1007/s10071-008-0185-9 18712531

[57] Baker JM, Shivik J, Jordan KE (2011) Tracking of food quantity by coyotes (*Canis latrans*). Behav Process 88(2): 72–75. 10.1016/j.beproc.2011.08.006 21856389

[58] Garland A, Low J, Burns KC (2012) Large quantities discriminations by North Island robins (*Petroica longipes*). Anim Cog 15(6): 1129–40. 10.1007/s10071-012-0537-3 22825034

[59] Jenkins HM, Moore BR (1973) The form of the auto-shaped response with food or water reinforcers. J Exp Anal Behav 20(2): 163–181. 4752087 10.1901/jeab.1973.20-163 PMC1334117

[60] Emmerton J, Delius JD (1993) Beyond sensation: Visual cognition in pigeons. In Zeigler HP and Bischof HJ (Eds.) Vision, Brain, and Behavior in Birds (377–390). Cambridge, MA: MIT Press.

[61] Davis H, Memmott J (1982) Counting behavior in animals: A critical evaluation. Psychol Bul 92: 547–571.

